# Supplementary material for: Drought and climate change impacts on cooling water shortages and electricity prices in Great Britain
Source: Nat Commun. 2020 May 7;11:2239. doi: 10.1038/s41467-020-16012-2 (PMC7206141; doi:10.1038/s41467-020-16012-2)
Supplement: Supplementary file 1 — Supplementary Information [file 41467_2020_16012_MOESM1_ESM.pdf]

Supplementary Information

Drought and climate change impacts on cooling water shortages and electricity prices in Great Britain

Byers et al.

Supplementary Figure 1

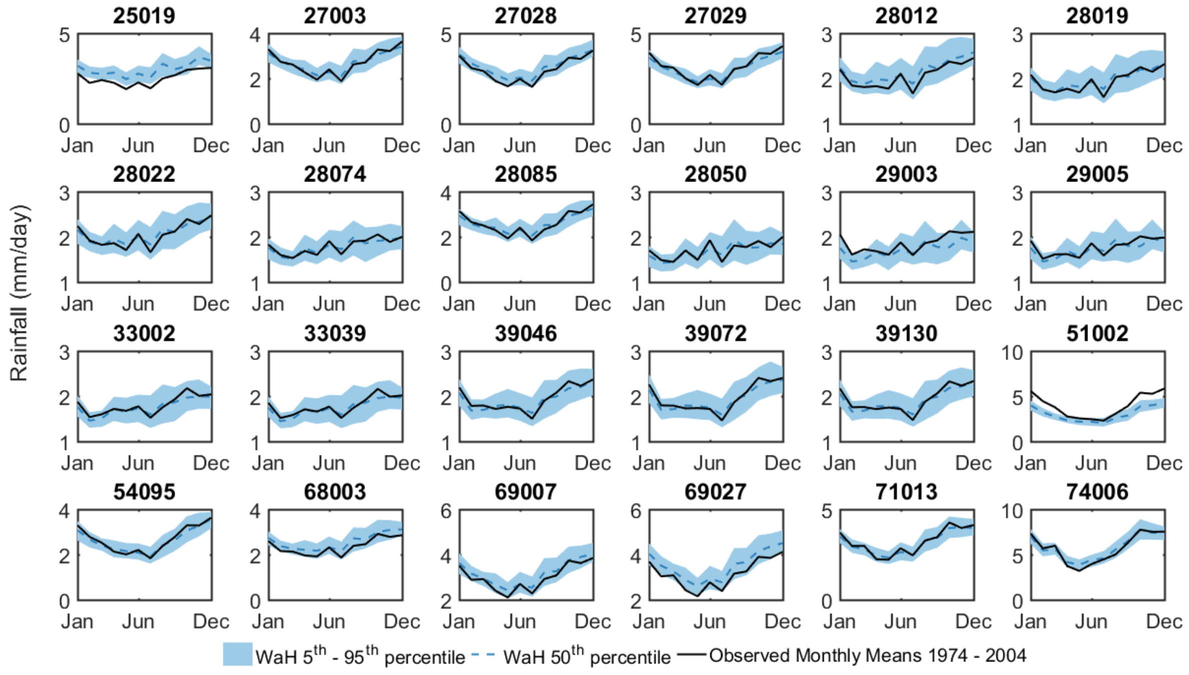

Hydrological calibration at each gauge. *Graphs showing the spread of precipitation for the W@H2 climate simulations for the Baseline period compared to the Observed record (black). N.B. wider blue bands do not represent error of the model but the internal (natural) variability of the climate.*

Supplementary Figure 2

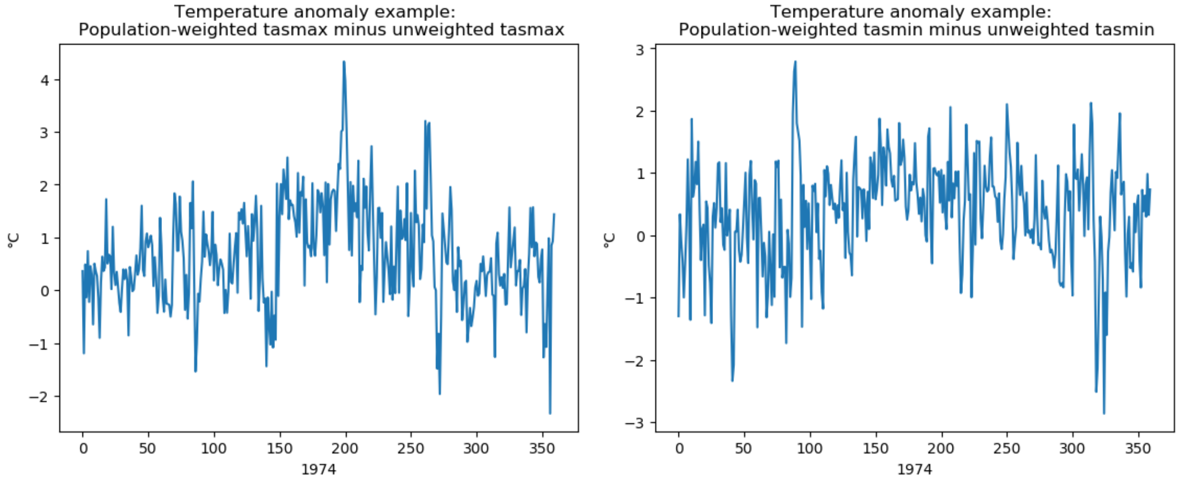

The effect of the population weighted temperature inputs. *Two example timeseries for one year showing the anomaly between the population-weighted average and unweighted average temperatures taken across the 13 MIDAS weather stations. Left shows 'tasmax', daily maximum air temperature, and right shows 'tasmin', daily minimum air temperature. This anomaly indicates the benefit of applying the population-weighting to the meteorological variables so as to improve the model calibration and reduce under- or over-estimation of the demand.*

Supplementary Figure 3

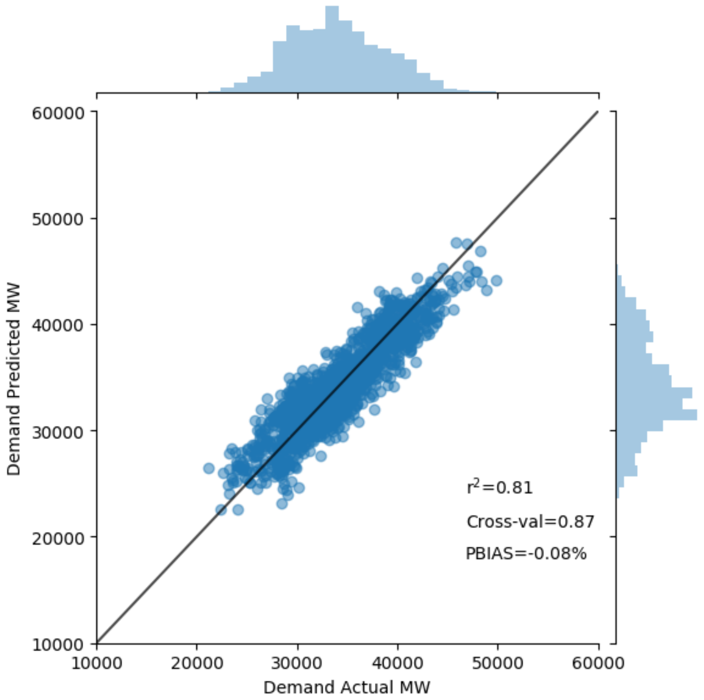

Scatter plot of the Predicted vs. Actual demand from the calibrated meteorological-demand model. *The model was set up and calibrated using the Python package scikit-learn. We used machine-learning gradient boosting regression trees algorithm [1, 2] with initial hyperparameters (estimators n=3000, max depth=3, min samples split = 2, learning rate 0.01, Huber loss function).*

Supplementary Figure 4

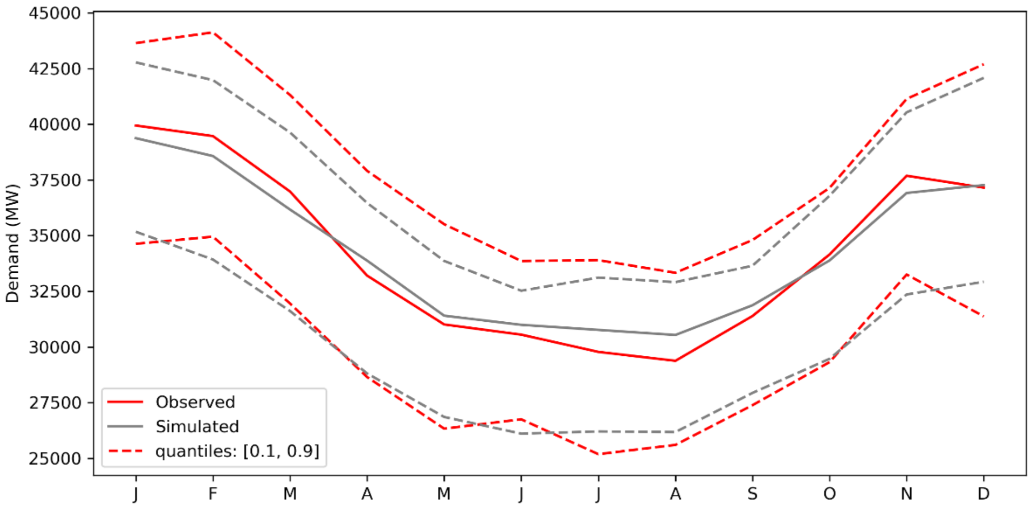

Seasonal profiles of demand. *For the median, 0.1 and 0.9 quantiles, to compare demands for the Observed period (2012-17) and as Simulated (30 years, Baseline climate) – from Supplementary Table 3.*

Supplementary Figure 5

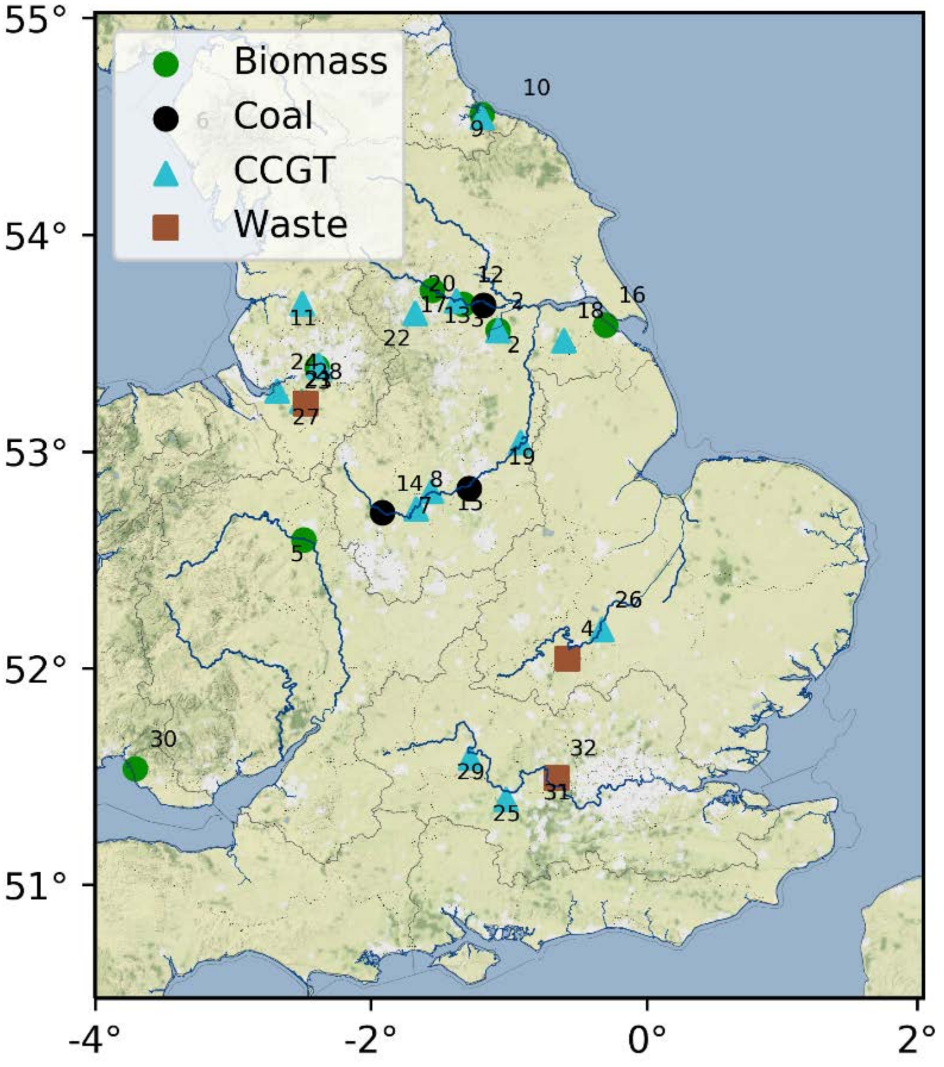

Map of power plants. *Map of thermal power plants and types in Great Britain used in this study. Label numbers correspond to the plants in Supplementary Table 4.*

Supplementary Figure 6

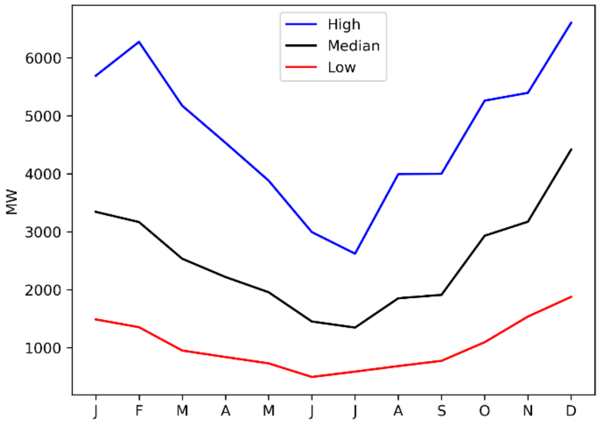

Monthly values for the combined renewables under 3 production scenarios. *Low (10th percentile), Median (50th) and High (90th).*

Supplementary Figure 7

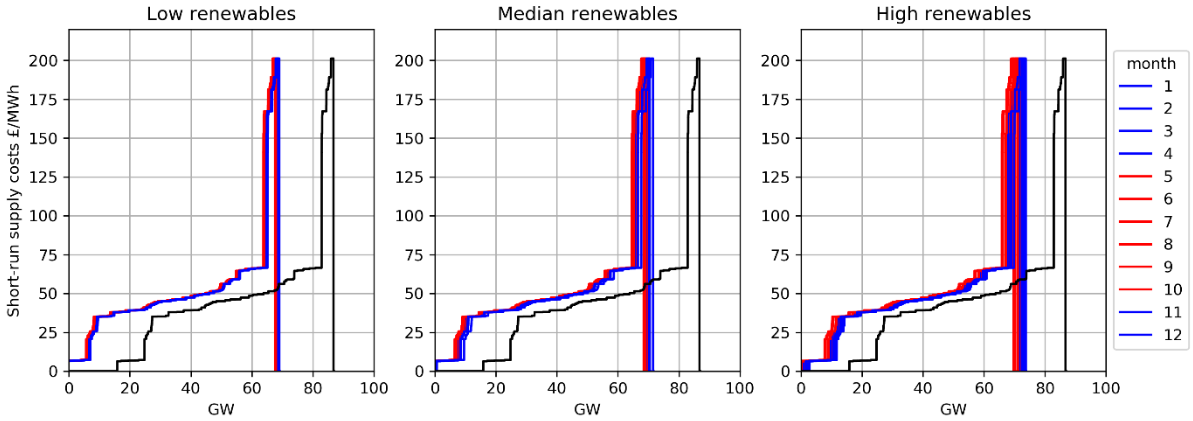

Monthly supply curves. *The monthly supply curves (summer/winter in red//blue) for the three renewables production scenarios, compared to the unadjusted full capacity supply curve in black. In the low scenario, there is little difference between monthly production, compared to the larger differences between summer and winter in the high production scenario.*

Supplementary Figure 8

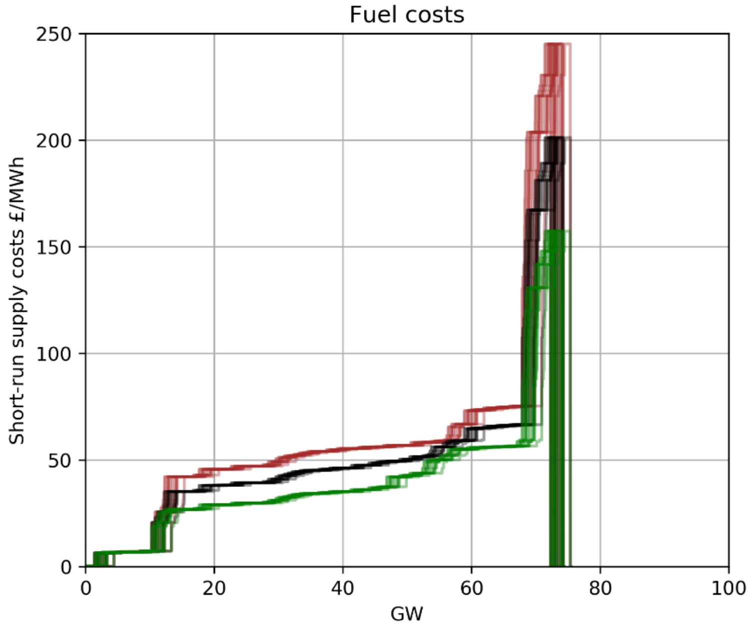

Supply curves under fuel costs scenarios. *Sensitivity to fuel prices was also tested by adjusting the fuel prices +/- 25% within the ELSI model as shown in Supplementary Table 5. Black lines are the monthly supply curves for the central scenario, Brown for the +25% (High) and Green for the -25% (Low).*

Supplementary Table 1

Soft-landing approach to environmental flow requirements and reductions. *In this study we incorporate the "soft landing" approach whereby water users gradually reduce withdrawals depending on the flow, as determined by the percentiles of the historical flow duration curve.*

Q	*99.9*	*99*	*98*	*97*	*96*	*95*	*94*	*93*	*92*	*91*	*90*
Permitted withdrawal %	0	10	20	30	40	50	60	70	80	90	100
Actual plant output %	0	0*	0*	30	40	50	60	70	80	90	100

** Reduced to 0 as uneconomic to operate plants as such low load.*

Supplementary Table 2

MIDAS stations and related population weighting *Urban areas, population and MIDAS weather station numbers used in study for Great Britain. To calibrate the meteorological demand model, the population-weighted average of observed weather variables was used, corresponding to the urban area pulations*[3] *and MIDAS weather stations below*[4]*.*

	Urban area	Population	MIDAS Station
1	Greater London Built-up Area	9,787,426	726
2	Greater Manchester Built-up Area	2,553,379	30690
3	West Midlands Built-up Area	2,440,986	19187
4	West Yorkshire Built-up Area	1,777,934	513
5	Greater Glasgow Built-up Area	1,209,143	24125
6	Liverpool Built-up Area	864,122	17309
7	South Hampshire Built-up Area	855,569	847
8	Tyneside Built-up Area	774,891	30523
9	Nottingham Built-up Area	729,977	556
10	Sheffield Built-up Area	685,368	56958
11	Bristol Built-up Area	617,280	692
12	Leicester Built-up Area	508,916	892
13	Edinburgh Built-up Area	482,005	19260

Supplementary Table 3

Percentage deviation values for each month and at different quantiles for the Baseline climate.

		quantile								
Month	Mean	0.1	0.2	0.3	0.4	0.5	0.6	0.7	0.8	0.9
1	-1.0	1.6	0.1	0.0	-1.9	-1.4	-1.9	-1.6	-1.2	-2.0
2	-3.2	-2.9	-1.2	-2.1	-2.0	-2.3	-2.9	-3.5	-4.6	-4.9
3	-2.1	-1.1	-0.6	-0.4	-2.0	-2.2	-2.1	-2.4	-2.8	-4.1
4	-0.3	0.6	0.0	2.6	2.3	2.0	1.2	-1.2	-2.7	-3.8
5	-0.8	2.0	0.3	3.1	2.4	1.3	-0.8	-3.1	-4.3	-4.6
6	-0.4	-2.4	-1.9	3.9	2.3	1.4	0.4	-2.2	-3.6	-3.9
7	-0.9	3.3	-3.5	-1.6	2.3	2.6	-0.9	-3.2	-3.9	-2.9
8	0.4	1.1	-0.5	3.4	4.2	2.9	0.9	-2.2	-3.3	-2.2
9	0.0	2.0	0.5	3.7	2.7	1.5	0.0	-2.4	-2.9	-3.3
10	0.1	0.5	0.9	0.9	-1.0	-0.8	-0.4	-0.2	-0.2	-1.0
11	-1.2	-2.7	-1.6	-1.0	-0.9	-2.1	-1.7	-1.5	-1.5	-1.5
12	1.1	5.0	1.9	2.3	2.2	0.3	0.0	-0.6	-0.1	-1.4

Supplementary Table 4

Power plants and corresponding gauges. *List of power plants considered in the study. CCGT is Combined Cycle Gas Turbine, MIW is Municipal and Industrial Waste Incineration, CHP is Combined Heat and Power. Subset of dataset [5, 6, 7] joined with gauge station information from [8].*

#	Site	Technology	MW_e	Cooling system	Region	Country	Gauge #	River	Station
1	Hatfield Park 2	Coal	450	Evaporative	Yorkshire & Humber	England	28050	Torne	Auckley
2	Hatfield Park 1	CCGT	450	Evaporative	Yorkshire & Humber	England	28050	Torne	Auckley
3	Eggborough	Coal	1960	Evaporative	Yorkshire & Humber	England	27003	Aire	Beal Weir
4	Rookery South	MIW	65	-	East	England	33002	Bedford Ouse	Bedford
5	Ironbridge	Biomass	900	Evaporative	West Midlands	England	54095	Severn	Buildwas
6	Fellside CHP	CCGT CHP	180	Hybrid	North West	England	74006	Calder	Calder Hall
7	Willington C	CCGT	2400	Evaporative	East Midlands	England	28085	Derwent	St Mary's Bridge
8	Drakelow	CCGT	1220	Evaporative	East Midlands	England	28019	Trent	Drakelow Park
9	Wilton	Coal-Biomass	150	Evaporative	North East	England	25019	Leven	Easby
10	Teeside	CCGT	45	Evaporative	North East	England	25019	Leven	Easby
11	Blackburn Mill	CCGT	60	Hybrid	North West	England	71013	Darwen	Ewood
12	Ferrybridge MFC	Biomass	68	Evaporative	Yorkshire & Humber	England	27003	Aire	Beal Weir
13	Castleford	CCGT	56	Once through	Yorkshire & Humber	England	27003	Aire	Beal Weir
14	Rugeley	Coal	1006	Evaporative	West Midlands	England	28002	Blithe	Hamstall Ridware
15	Ratcliffe	Coal	1960	Evaporative	East Midlands	England	28074	Soar	Kegworth
16	Stallingborough	Biomass	65	Evaporative	East Midlands	England	29001	Waithe Beck	Brigsley
17	Pollington	Biomass	53	Evaporative	Yorkshire & Humber	England	27028	Aire	Armley
18	Glanford Brigg	CCGT	260	Evaporative	Yorkshire & Humber	England	29005	Rase	Bishopbridge
19	Staythorpe C	CCGT	1724	Evaporative	East Midlands	England	28022	Trent	North Muskham
20	Thornhill	CCGT	50	Once through	Yorkshire & Humber	England	27074	Spen Beck	Northorpe
21	Bridestones Carrington	CCGT	860	Evaporative	West Midlands	England	69013	Sinderland Brook	Partington
22	Carrington	CCGT	380	Evaporative	West Midlands	England	69013	Sinderland Brook	Partington
23	SAICA Paper Mill	Biomass	42	Evaporative	North West	England	69013	Sinderland Brook	Partington
24	Rocksavage	CCGT	810	Evaporative	North West	England	68003	Dane	Rudheath
25	Burghfield	CCGT	47	Open loop	South East	England	39130	Thames	Reading
26	Little Barford	CCGT	714	Evaporative	East	England	33039	Bedford Ouse	Roxton
27	Lostock	MIW	60	Evaporative	North West	England	68003	Dane	Rudheath
28	Sandbach	CCGT	50	Evaporative	North West	England	68003	Dane	Rudheath
29	Didcot B	CCGT	1430	Hybrid	South East	England	39046	Thames	Sutton Courtenay
30	Western Wood	Biomass	35	Evaporative	Wales	Wales	51002	Horner Water	West Luccombe
31	Slough	Biomass	61	Evaporative	South East	England	39072	Thames	Royal Windsor Park
32	Fibrepower	MIW	50	Evaporative	South East	England	39072	Thames	Royal Windsor Park

Supplementary Table 5

Short-run marginal costs for the powerplants in the model.

	SRMC £/MWh	SRMC-LOW £/MWh	SRMC-HIGH £/MWh	Relation to ELSI SRMC costs
Hydro	0	0	0	
Hydro 5-16MW	0	0	0	Hydro
Solar (<10kW)	0	0	0	
Solar (250-1000kW)	0	0	0	
Solar (large)	0	0	0	
Solar (small)	0	0	0	
Wind	0	0	0	
Wind (<50kW)	0	0	0	
Wind (100-1500kW)	0	0	0	
Wind (offshore) R2	0	0	0	
Wind (offshore) R3	0	0	0	
Nuclear	6.5	6.5	6.5	
Biomass	25	25	25	
Straw	25	25	25	Oil
Biomass (dedicated, <50 MW)	27.71	22.22	33.21	CHP
Biomass CHP	27.71	22.22	33.21	CHP
Meat & bone meal	27.71	22.22	33.21	Oil
Waste	27.71	22.22	33.21	Oil
Waste CHP	27.71	22.22	33.21	CHP
coal / biomass (conversion)	40.81	36.53	44.525	Average of Coal & Oil
Gas	43.01	34.47	51.54	Gas - Other
CCGT	45.35	34.47	54.35	
coal	56.61	48.06	64.05	
Hydro / pumped storage	56.61	56.61	56.61	Pumped Storage Gen
Pumped storage	56.61	56.61	56.61	Pumped Storage Gen
Coal / oil	93.34	75.17	111.02	Average of Coal & Biomass
Diesel	130.14	102.28	157.99	Oil
Gas / oil	130.14	102.28	157.99	Oil
Gas oil	130.14	102.28	157.99	Oil
Gas oil / kerosene	130.14	102.28	157.99	Oil
Light oil	130.14	102.28	157.99	Oil
OCGT 100MW	171.17	133.84	208.51	OCGT
OCGT 200MW	171.17	133.84	208.51	OCGT
OCGT 400MW	171.17	133.84	208.51	OCGT
OCGT 600MW	171.17	133.84	208.51	OCGT

Supplementary Note 1

Daily generation data from 2015-2016 of electricity production [9] from hydro, pumped storage, wind and solar was used to develop monthly values of combined renewables electricity generation. Using daily sum of sources, the 10th, 50th and 90th percentile values for each month are used to represent the low, median and high renewables generation values. This method, similarly used by Pöyry, UK for the Department for Business, Energy and Industrial Strategy (BEIS) in the Dynamic Demand Model (DDM) and National Grid in the Electricity Scenario Illustrator provides a statistical method to sample wind production uncertainty without running a full ensemble of wind simulation and production runs. Short-run marginal costs (SRMC) are derived from the National Grid ELSI model [10, 11] which is a power market economic dispatch model that assesses optimal dispatch through unconstrained and constrained dispatches. ELSI costs for 2017 were used to match the powerplants dataset.

To represent wider range of short-run generation costs between power plants of the same technology, variance was added according to the age of the units, such that newer (and more efficient) units would have marginally lower operational costs than older plants.

For all thermal plants in the UK (n=134)[5], for which many of the sites have been operation for decades, we searched the internet for information on major powerplant upgrades, for example replacement turbine units, in which case the upgrade year was noted.

For each technology, parameter, s, defines the spread of short run generation cost[12], as a fraction, around the central estimate short run generation cost, c, that occurs halfway through the lifetime of the plant, y_l. Therefore the maximum (minimum) short run cost and end (start) of a plant's life is:

$$c_{max} = c + \frac{c \cdot s}{2}$$

$$c_{min} = c - \frac{c \cdot s}{2}$$

Thus the generation cost c_n, which increases linearly, in the current year y_n is found by:

$$c_n = (c_{max} - c_{\min}) \cdot \frac{y_n}{y_l} + c_{min} + i$$

Spread parameter s was chosen based on analysis of Ofgem spark (gas) and dark (coal) spreads cost data [13] between the low and high efficiency spark costs. i is a randomly chosen residual value in the range of 0-1 for cases to differentiate where two plants have exactly the same costs so that they do not overlap in the supply curve. Note that once the short-run costs were defined, there were not updated through time during the 30-year timeseries simulations, so as not to change the cost distributions within a time period.

Supplementary References

1. Pedregosa F*, et al.* Scikit-learn: Machine learning in Python. *Journal of machine learning research* **12**, 2825-2830 (2011).

2. Friedman JH. Greedy function approximation: a gradient boosting machine. *Annals of statistics*, 1189-1232 (2001).

3. UK Office for National Statistics. UNITED KINGDOM: Countries and Major Urban Areas. (ed ONS) (2011).

4. Met Office. *Met Office Integrated Data Archive System (MIDAS) Land and Marine Surface Stations Data (1853-current)* (2012).

5. BEIS. Power stations in the United Kingdom as at May 2017. In: *Digest of UK Energy Statistics (DUKES)*) (2017).

6. Byers EA, Hall JW, Amezaga JM. Electricity generation and cooling water use: UK pathways to 2050. *Global Environmental Change* **25**, 16-30 (2014).

7. Byers EA*, et al.* Cooling water for Britain's future electricity supply. *Proceedings of the Institution of Civil Engineers - Energy* **168**, 188-204 (2015).

8. Centre for Ecology & Hydrology. National River Flow Archive.) (2018).

9. Gridwatch. *G.B. National Grid Status* (2018).

10. National Grid. *ELSI User Guide* (2018).

11. National Grid. Electricity Scenario Illustrator Excel workbook. (ed National Grid) (2018).

12. BEIS. *Electricity Generation Costs*. Department for Business, Energy and Industrial Strategy (2016).

13. Ofgem. *Spark and dark spreads (GB)*. Ofgem (2018).
